# Sensory input to cortex encoded on low-dimensional periphery-correlated subspaces

**DOI:** 10.1093/pnasnexus/pgae010

**Published:** 2024-01-10

**Authors:** Andrea K Barreiro, Antonio J Fontenele, Cheng Ly, Prashant C Raju, Shree Hari Gautam, Woodrow L Shew

**Affiliations:** Department of Mathematics, Southern Methodist University, Dallas, TX 75275, USA; Department of Physics, UA Integrative Systems Neuroscience, University of Arkansas, Fayetteville, AR 72701, USA; Department of Statistical Sciences and Operations Research, Virginia Commonwealth University, Richmond, VA 23284, USA; Department of Physics, UA Integrative Systems Neuroscience, University of Arkansas, Fayetteville, AR 72701, USA; Department of Physics, UA Integrative Systems Neuroscience, University of Arkansas, Fayetteville, AR 72701, USA; Department of Physics, UA Integrative Systems Neuroscience, University of Arkansas, Fayetteville, AR 72701, USA

## Abstract

As information about the world is conveyed from the sensory periphery to central neural circuits, it mixes with complex ongoing cortical activity. How do neural populations keep track of sensory signals, separating them from noisy ongoing activity? Here, we show that sensory signals are encoded more reliably in certain low-dimensional subspaces. These coding subspaces are defined by correlations between neural activity in the primary sensory cortex and upstream sensory brain regions; the most correlated dimensions were best for decoding. We analytically show that these correlation-based coding subspaces improve, reaching optimal limits (without an ideal observer), as noise correlations between cortex and upstream regions are reduced. We show that this principle generalizes across diverse sensory stimuli in the olfactory system and the visual system of awake mice. Our results demonstrate an algorithm the cortex may use to multiplex different functions, processing sensory input in low-dimensional subspaces separate from other ongoing functions.

**Significance Statement**

Traditionally, primary sensory cortex was thought to have one job—processing sensory signals. As technical advances allow more holistic measurements of the brain and body in action, it has become clear that the primary sensory cortex is involved with many other aspects of brain function, not just dealing with sensory input. How can a single neural circuit juggle multiple jobs simultaneously? Here, we use numerical, analytical, and experimental methods to demonstrate an algorithm the brain may use to solve this problem by separating different jobs into different subspaces defined by correlations between the primary sensory cortex and the brain regions that source the sensory input signals.

## Introduction

Neurons in primary sensory cortices are involved in diverse aspects of brain function; their activity is not limited to encoding sensory signals (1–4). It is becoming increasingly clear that the primary sensory cortex is a multiplex, full of cross-talk and multipurpose signals. For example, neuronal activity in the primary visual cortex (V1) does not just encode physical features of visual stimuli, but is also related to locomotion (5, 6), whisking and pupil diameter (7), forepaw manipulations (8), decision making (8–10), and learned consequences (rewards) of the visual stimuli (11). Similarly, neurons in the primary olfactory cortex (piriform cortex, PC) go beyond odor coding, exhibiting activity related to spatial navigation (12), thirst (13), decision making (10), and working memory (14), and can drive distinct behaviors (15). In general, involvement in these diverse “nonsensory” functions will vary across repeated trials of a sensory stimulus. Thus, it is not surprising that the responses of single cortical neurons to a repeated sensory stimulus vary greatly from trial-to-trial, often making the stimulus identity impossible to decode accurately with a single neuron. How does the brain reliably keep track of sensory signals when they are mixed into the complex, multipurpose dynamics of the cortex?

Here we propose a population-level solution to this problem. We start from the fact that at the sensory periphery, neuronal activity is purely sensory and not mixed with other functions. As the signal traverses the sensory hierarchy from the periphery to the cortex, it becomes increasingly mixed with nonsensory signals due to increasingly recurrent interactions with other brain regions (16–18). It stands to reason that sensory signals in thalamic nuclei or olfactory bulb (OB) could be less noisy (closer to purely sensory) than sensory signals in cortex. Consistent with this, the dorsal lateral geniculate nucleus (LGN), which provides input to V1, exhibits a response to visual stimuli that has lower dimensionality than V1 (19) and is less affected by locomotion than response in V1 (6). Similarly, LGN firing is modulated more by sensory input and less by behavioral context compared to V1 (16). Likewise, OB, which innervates PC, is often less noisy than PC. For example, OB has more neurons that are clearly responsive to olfactory stimuli compared with PC (20). (In Fig. S1, we directly show that the population-level signal-to-noise ratio is greater in OB compared to PC and greater in LGN compared to V1 for the data analyzed further below.) Thus, we hypothesized that certain coding subspaces in the cortex—those that share variability with subspaces in upstream sensory regions (thalamus or OB)—may contain sensory signals with less noise.

What do we mean by a coding subspace? Considering a population, rather than single neurons (21, 22), the single-trial response of *N* cortical neurons is a vector in an *N*-dimensional space; e.g. the sixth component of the vector is the response of the sixth neuron, and so on. The responses to many repeated trials of two different stimuli can be represented as two clouds of points in *N*-dimensional cortical space, one point for each trial, and one cloud for each stimulus type. The spread of each cloud of points reflects the trial-to-trial variability (the nonsensory signals discussed above) and the overlap of the two clouds makes decoding the stimuli difficult. However, if the response variability due to nonsensory noise lies along different directions than the variability due to switching the sensory signal, then decoding can be greatly enhanced by projecting the *N*-dimensional response onto a coding subspace, i.e. a lower dimensional subspace that excludes some noise. Our hypothesis here is that such coding subspaces can be found by considering signal correlations and noise correlations between the cortical population and upstream populations.

Recent studies have adopted conceptually related approaches demonstrating that high-dimensional neural circuits may manage multiple operations by performing them in different subspaces. For instance, neurons in the mouse auditory cortex “rotate” sensory representations from a sensory subspace to a memory subspace over time (23). Neurons in rat posterior parietal cortex use different subspaces to represent decision and movement (24). Motor preparatory activity in monkeys contains a subspace that does not impact movement, i.e. in a “nullspace” (25). Monkeys making a choice about motion and color of a visual stimulus exhibited neurons in prefrontal cortex (PFC) that used three different subspaces to encode color, motion, and choice (26). Similarly, working memory and movement planning are separated into different subspaces within a population of PFC neurons in monkeys (27). In anesthetized monkeys, signals are transmitted between V1 and V2 in a “communication subspace” (28). The outputs of mouse cerebellar neurons were shown to represent quiescent and active behavioral states in orthogonal subspaces (29). Computational models together with human brain imaging suggest that orthogonal subspaces are used to represent different task variables in an image classification task (30). Computational models of entorhinal cortex suggest that encoding of spatial navigation and navigation-independent context are separated into orthogonal subspaces (31). Our work here extends these ideas, establishing sensory subspaces in cortex and in the sensory brain regions that provide input to cortex and an algorithm for finding these subspaces.

Projecting high-dimensional activity into a lower dimensional coding subspace is a type of dimensionality reduction. More generally, dimensionality reduction has long been recognized and used to improve decoding of sensory signals with supervised pattern classification techniques like linear discriminant analysis (LDA) (32, 33). However, LDA and similar techniques require semantic labels for the stimuli, which can be challenging to implement in a biologically plausible way (but perhaps not impossible, see e.g. Refs. (34, 35)). Here, we identify a totally unsupervised decoding strategy. We show that low-dimensional, optimal coding subspaces can be found without any knowledge of stimuli identities by considering correlations between cortex and upstream brain areas that provide input to cortex. Using canonical correlation analysis (CCA, see Refs. (22, 36) for an introduction), we define subspaces in cortex and subspaces in LGN or OB in which responses to stimuli are most correlated across the brain regions. We show that these cross-population correlated subspaces can effectively separate signal from noise, often approaching the theoretical limits of optimal decoders (like LDA). We developed an analytical approach to better understand these coding subspaces and successfully predicted improved coding subspaces among neurons with low cross-population noise correlations. We first present the theory and then test its predictions using spike data.

## Results

**Fig. 1.**
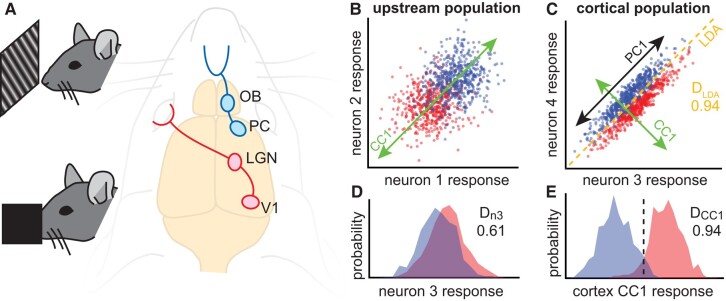
Using inter-regional correlations to find coding subspace. A) We hypothesize that correlations between primary sensory cortices (PC or V1) and upstream populations (OB or LGN) can be used (via CCA) to identify coding subspaces with reduced noise. We test this hypothesis in awake mice using simultaneous recordings from V1 and LGN during visual stimulation (top) or PC and OB during olfactory stimulation (bottom). B) Each point represents the simulated response of neurons 1 and 2 to one of two hypothetical stimuli (red and blue). C) Simulated responses of neurons 3 and 4 in the cortical population. The optimal LDA decoder achieves 94% accuracy. CCA applied to the two populations identifies a linear subspace (CC1, green) for each population. Projection of each population's response onto its CC1 results in maximized correlation across populations. PCA finds the linear subspace (PC1, black) with maximal variability; in this case, the variability is due to noise. D) Response distributions for the two stimulus types overlap substantially for neuron 3, resulting in suboptimal decoding accuracy (61%). E) When projected onto CC1, the response distributions better separate the two stimulus types, achieving optimal decoding accuracy (94%, same as LDA). The dashed line indicates the optimal threshold used for calculating the decoding accuracy.

A central idea underpinning our theory is that the brain can improve decoding of sensory input by projecting neural activity onto a subspace which excludes some of the “noise” that compromises decoding. We hypothesized that we could identify such decoding subspaces based on inter-regional correlations between cortex (V1 or PC) and upstream extracortical regions (LGN or OB). To demonstrate how this might work, we first present a simple, instructive case based on simulated data: two neurons in cortex (Fig. 1C) and two neurons in the upstream region (Fig. 1B). In this simulated example, the responses are drawn from a multivariate Gaussian distribution (Materials and methods) with parameters chosen such that the two cortical neurons have strong noise correlations and a small difference in mean response for the two stimuli. The two extracortical neurons have noisy overlapping responses to the two stimuli. (In this example, there are no cross-population noise correlations, which is important for our approach, as we discuss further below.) All four of these neurons are rather poor decoders at the single neuron level, but decoding improves substantially when projected onto a particular subspace (green lines in Fig. 1B and C). The optimal subspace can easily be found using LDA (the dashed line in Fig. 1C is the LDA classification boundary), but LDA requires knowledge of the stimulus identities; the brain does not have direct access to stimulus identities before they are decoded. The optimal subspace can also be found, without knowledge of stimulus identity, by performing CCA, which is the key advance presented in this paper.

Before proceeding, we briefly introduce CCA for unfamiliar readers (see also Refs. (22, 36)), comparing and contrasting with the more commonly used principal component analysis (PCA). Similar to PCA, CCA generates a set of basis vectors based on the covariance matrix of multivariate data; these are the canonical components (CCs) for CCA and the principal components (PCs) for PCA. In our context, PCA would take a set of spike count responses from a single population of neurons and generate one set of components. In contrast, two sets of spike count responses, one from each of two different populations of neurons, are the inputs to CCA. Likewise, CCA generates two sets of components, one for each population. The first canonical component (CC1) for the first population is related to CC1 for the second population; CCA is defined such that the correlation between the two populations is maximized when they are projected onto their respective CC1s. In contrast, PCA is defined such that projection onto PC1 maximizes variance. In the example in Fig. 1C, PC1 (black arrow) is aligned with noise fluctuations in the cortical population, but CC1 (green arrow) is aligned with the direction along which signal varies most (orthogonal to PC1 in this case), thus identifying the optimal decoding subspace (orthogonal to the LDA classification boundary). In general, PCA will not identify the optimal decoding subspace.

Throughout this paper, we report various types of decoding accuracy including *D*_LDA_, *D*_n3_, *D*_CC1_, and optimal *D*. In all of these cases, we are working with a 1D response to two stimuli; it is 1D because it is projected onto a line or, in the case of *D*_n3_, simply because it is the response of one single neuron. We define decoding accuracy as the fraction of correctly predicted stimuli using the best possible threshold to separate the responses. For example, in Fig. 1E, the best possible threshold is marked with the dashed line.

**Fig. 2.**
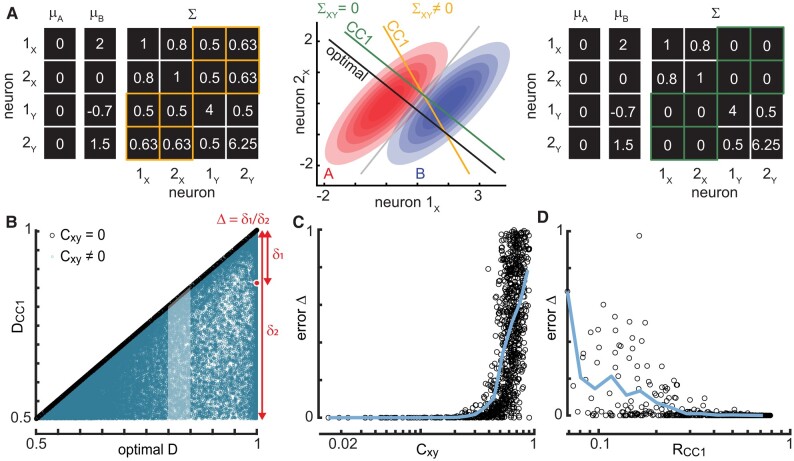
Cross-population noise correlations and CC1 correlation determine the accuracy of coding subspace. A) Example parameters for population *x* (neurons 1*_x_* and 2*_x_*) and population *y* (neurons 1*_y_* and 2*_y_*) for two stimulus types (A and B). When stimulus-independent cross-population covariance between populations *x* and *y* is zero (green), the first component CC1 is the same as the optimal decoding dimension. Nonzero cross-population covariance results in deviations from the optimal decoding dimension. B) Each point represents one of 50,000 randomly chosen parameter sets for the 2 × 2 population. *D*_CC1_ is perfect (same as optimal *D*, Δ = 0) if the cross-population noise correlations are zero (black points). If cross-population noise correlations are nonzero, then CC1 decoding is often suboptimal (blue points, Δ > 0). The highlighted set of blue points is studied further in panels C and D. C) Decoding error (Δ) is the normalized distance from optimal *D* (see red diagram in panel B). *D*_CC1_ is near-optimal (low Δ) for *C_xy_* below about 0.4. The points shown in panel C exclude cases with *R*_CC1_ < 0.75 (Materials and methods). D) Achieving low decoding error Δ also requires sufficiently large *R*_CC1_. Shown results exclude cases with *C_xy_* > 0.1 (Materials and methods). Blue lines in panels C and D—moving average of points.

Is the example in Fig. 1 indicative of a more general principle? Does CC1 always reveal the optimal decoding subspace? To answer these questions, we next performed a more extensive analytical and numerical study of this 2 × 2 case considering a wide variety of correlations among the four neurons and signal-to-noise scenarios. We assumed that each neuron had responses to two different stimuli that were drawn from multivariate Gaussian distributions. We further assumed that the responses of the 4 neurons are governed by 15 parameters: 8 mean responses (2 stimuli × 4 means), 4 variances, 2 within-population covariances, and 1 cross-population covariance (Fig. 2A). Variances and covariances were assumed to be the same for the two stimuli. By centering on the mean response for one stimulus type, we reduce this to 11 parameters, without loss of generality. We considered 50,000 different configurations of these 11 parameters, drawn randomly (Materials and methods). We note that here we used analytical methods to compute the CC directions, optimal *D*, and other quantities; these results depend only on the 11 parameters discussed above and are not limited by finite numbers of samples (Materials and methods).

We found that CC1 is not, in general, well-aligned with the optimal decoding subspace, resulting in decoding *D*_CC1_ that is often suboptimal (Fig. 2B, blue). Nonetheless, for many of the 50,000 random populations, *D*_CC1_ was very close to optimal. Next, we asked what factors determine whether the subspace defined by CC1 is near-optimal or not? We found that the most important factor was correlated noise shared across the two populations. This is consistent with previous work highlighting the importance of noise correlations for decoding (37, 38). When we set these cross-population noise correlations to zero, keeping all the other parameters fixed, the CC1 direction was exactly optimal in all cases (Fig. 2B, black and Supplementary material [Mathematical Results]). Thus, our theory predicts that if there were no stimulus-independent shared variability between, say, LGN and V1 neurons, then CCA would be a perfect algorithm for visual decoding, even without knowing the stimulus labels. But, of course, it is very unlikely that LGN and V1 have zero noise correlations. Therefore, we next asked whether near-optimal CC1 decoding might be achieved even with small, nonzero noise correlations? Or does sensitivity to noise correlations render the algorithm useless in real neural systems? To answer these questions, we examined how *D*_CC1_ depends on cross-population noise correlations *C_xy_*. We found that nonzero, but small *C_xy_*—below about 0.4 for our simulations—resulted in a CC1 that was very close to the optimal subspace (Fig. 2C). Thus, the algorithm is robust to some degree of noise correlations and could be useful for real sensory systems.

One further prediction from the theory comes from considering the correlation coefficient *R*_CC1_ of the responses across the two populations after projection onto the two CC1 directions. *R*_CC1_ is the quantity that is maximized by the CCA algorithm. If noise correlations are small, then we expect CCA to identify a CC1 direction that aligns with signal fluctuations and *R*_CC1_ should reflect the strength of the signal. We confirmed this; for low *C_xy_* cases, we found that if *R*_CC1_ is too low, then CC1 decoding deteriorates (Fig. 2D). In this case, the two populations are nearly independent with no shared information about noise or signal. Thus, a more complete prediction from our theory is as follows. If there exist neurons in the primary sensory cortex and the upstream brain regions that have sufficiently low *C_xy_* and sufficiently high *R*_CC1_, then the sensory system could identify low-noise coding subspaces using CCA. Next, we set out to test this prediction in real neurons recorded in awake mice.

Before proceeding, let us examine what it could mean for the experiments to *not* confirm the theory. Why might our predicted CCA decoding subspaces not exist in reality? The most likely reason, as already discussed, is that cross-population noise correlations (*C_xy_*) could be too large. However, the theory could also fail, because the theory assumed (rather unrealistically) that the responses are Gaussian-distributed and that the neural covariance structure is the same for both stimuli (Materials and methods). In the experiments, spike count responses are nearly Poisson-distributed and many neurons will certainly have different variances for different stimuli. Finally, it is possible that the limited number of trials in experiments will preclude an effective test of the theory. This would not contradict the theory, but like LDA and other decoding schemes, our CCA decoding scheme when applied to real data with small numbers of trials can, in principle, provide overestimates of decoding accuracy. We will address each of these possible pitfalls below.

To test our predictions, we required simultaneous recordings from at least four neurons, two in the primary sensory cortex and two more in an upstream brain region that provides input to cortex. However, considering that many neurons may have substantial noise correlations across populations, our theory predicts that many neurons will be unsuitable for CCA decoding. Therefore, we sought out recordings with far more than two neurons in each region. We analyzed two datasets—one in the visual system (V1 and LGN), generated by the Allen Institute (16), and another in the olfactory system (PC and OB) generated by Bolding and Franks (20, 39). In the Bolding–Franks data (*n* = 8 mice), the number of recorded neurons per recording was 27 ± 6 (mean ± SD) in OB and 48 ± 11 in PC. We analyzed responses to 6 different odorants (fixed concentration) with 15 trials each. In the Allen Institute data (*n* = 9 mice), the number of recorded neurons per recording was 61 ± 27 in LGN and 245 ± 48 in V1. We analyzed responses to static gratings with 6 different orientations and 5 different spatial frequencies with 42 trials each. For both the olfactory and visual data, we defined response as the spike count in the 250 ms period following stimulus onset.

**Fig. 3.**
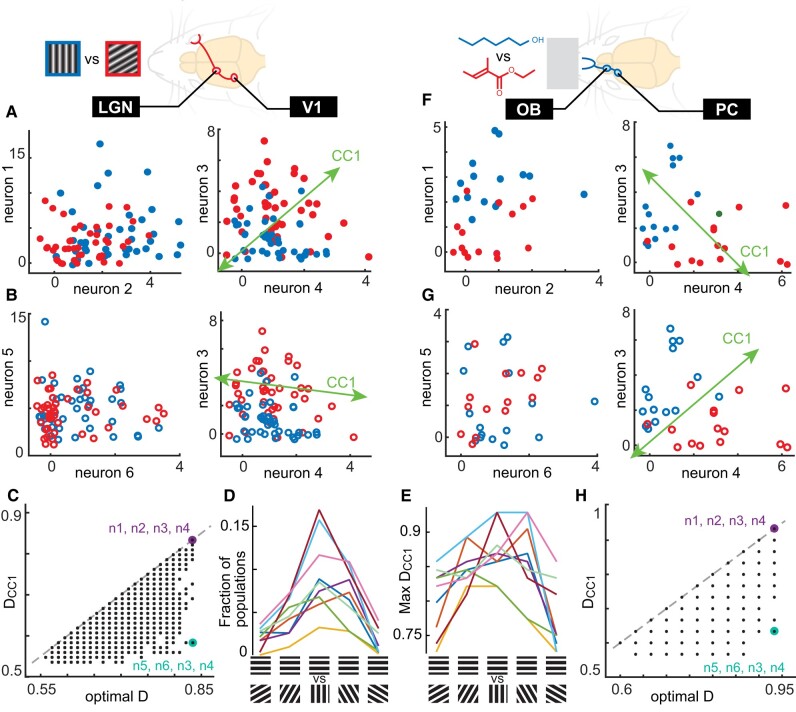
Dual-region neural populations achieve optimal CCA decoding of visual and olfactory stimuli. A) Spike count responses from two example neurons in LGN (left) and two in V1 (right), recorded from an awake mouse viewing two static gratings (90° and 30° orientations, 42 trials of each). Projection onto CC1 results in excellent (optimal) decoding in this example. B) Same as panel A, except with a different pair of LGN neurons. For this LGN population, the CC1 subspace in the cortex becomes suboptimal for decoding. C) Summary of CC1 decoding accuracy vs. optimal decoding for 10,000 randomly chosen 2 × 2 populations. Purple and cyan points indicate the examples shown in A and B, respectively. D) The fraction of all 10,000 populations with *D*_CC1_ above 0.7 is greatest when the angle difference between the two gratings is greatest. Each line represents a different mouse. E) Comparing all 10,000 populations, the maximum *D*_CC1_ was above 85% for 7 of 9 mice and typically for gratings with large angle differences. F–H) Same as panels A–C, but for a different awake mouse breathing two odorants (1-hexanol and ethyl tiglate, 15 trials of each). Note that the discrete values of *D* in panels C and H are determined by the number of stimulus trials; many points overlap at the same *D* value.

We begin with an example population from the visual system (two LGN neurons + two V1 neurons, Fig. 3A) and another example from the olfactory system (two OB neurons + two PC neurons, Fig. 3F). For both these examples, projecting the response onto CC1 results in optimal decoding for the cortex population. Here, optimal decoding is determined with a brute-force algorithm; we try all possible lines (with *π*/200 angular resolution), project responses onto each line, and pick the one with the highest decoding accuracy (Materials and methods). However, considering the same cortical population and choosing a different pair of neurons from LGN (Fig. 3B) or a different pair from OB (Fig. 3G) can result in a CC1 direction that is far from optimal. Going beyond these example cases, for each mouse and each pair of stimulus types, we considered 10,000 randomly chosen populations, each with 2 neurons in cortex and 2 neurons in the upstream region. For each such 2 × 2 population, we computed the decoding accuracy *D*_CC1_ for responses projected onto CC1. For the visual system data, *D*_CC1_ tended to be higher when decoding gratings with a greater angle difference; up to 15% of populations had *D*_CC1_ > 0.7 (Fig. 3D) and the maximum values of *D*_CC1_ exceeded 85% for most mice (Fig. 3E). When we compared *D*_CC1_ to optimal decoding (Fig. 3C and H), we found it was close to optimal for some populations, but more typically was far from optimal. Our theory suggests that the populations deviate from optimal for two reasons. First, they may have large cross-population noise correlations (*C_xy_*). Second, if *C_xy_* is not large, low *R*_CC1_ could cause deviation from optimal (Fig. 2). Before testing these predictions, we first verified that our measured values of *D*_CC1_ are statistically robust (i.e. not artificially high due to overfitting finite numbers of trials) using a 10-fold cross-validation method (Fig. S2).

To test whether our observed differences between *D*_CC1_ and optimal decoding were due to high *C_xy_* or low *R*_CC1_, we performed an additional analysis of the populations with high optimal decoding. Our rationale was that these cases can have the largest range of different possible deviations from optimal, and, thus, would be good candidates for studying the source of such deviations. For each mouse and stimulus pair, we selected the 50 populations (out of the 10,000 randomly chosen populations) with the greatest optimal decoding for the 2 cortical neurons. For each of these, we asked how *D*_CC1_ for the cortical population varied due to changing the upstream population. We tried 200 randomly chosen upstream populations for each cortical population. In this way, we kept optimal decoding accuracy fixed while varying *D*_CC1_, *C_xy_*, and *R*_CC1_.

**Fig. 4.**
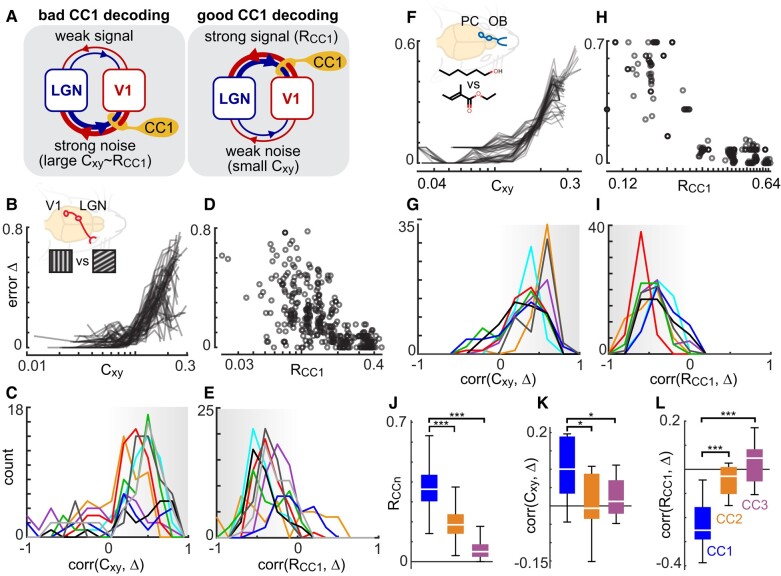
Explaining deviations from optimal CC1 decoding of visual and olfactory stimuli. A) CC1 decoding should perform poorly if the largest correlations between V1 and LGN, for example, are due to “noise,” i.e. not related to the sensory signal we want to decode. In this “bad CC1 decoding” case (left), CCA will identify a CC1 direction that is due to the noise correlation and *R*_CC1_ will reveal the strength of that noise correlation. When *C_xy_* is small enough, *R*_CC1_ will reflect the strength of signal, and CC1 will decode well (right). B) As predicted, the difference (error Δ) between optimal decoding and *D*_CC1_ was often strongly correlated with cross-population noise correlation *C_xy_*. Each line represents an average of over 200 different pairs of LGN neurons and one pair of V1 neurons (excluding those with small *R*_CC1_, see Materials and methods). C) Each distribution summarizes the correlations between error Δ and *C_xy_* for 200 populations in one mouse and one pair of stimuli (the color code for specific mouse/stimulus is shown in Fig. S1). Notice that most of these correlations are positive, like the examples in panel A. D) As predicted, error Δ is negatively correlated with *R*_CC1_. Each point represents one 2 × 2 population (excluding cases with large *C_xy_*, see Materials and methods). E) Each distribution summarizes the anticorrelations between error Δ and *R*_CC1_ for one mouse and stimulus pair (same cases as panel B). F–I) Same as panels A–D, but for OB + PC populations in different mice with olfactory stimulation. J) For 3 × 3 populations with high optimal decoding (Materials and methods), we show how *R*_CCn_ decreases for canonical components beyond the first. K) Only CC1 has a significant relationship between *C_xy_* and decoding error Δ. L) Only CC1 has a significant relationship between *R*_CC1_ and Δ.

Motivated by our theory (Fig. 2B–D), our working hypothesis for how *C_xy_*, and *R*_CC1_ impact *D*_CC1_ is illustrated conceptually in Fig. 4A. We consider that multiple (at least two) channels of interaction exist between, say V1 and LGN. For simplicity, we call one channel “signal,” which carries the sensory signals we wish to decode, and call the other channel “noise,” which has nothing to do with the sensory signals. Then, our working hypothesis is that *D*_CC1_ deviates from optimal when the “noise” channel dominates the inter-regional correlations. In this case *C_xy_*, will be large and CCA will, by definition, identify a CC1 direction that aligns with those noise fluctuations, *D*_CC1_ will be far from optimal, and *R*_CC1_ will indicate the strength of noise, not the strength of signal. In the opposite scenario (small *C_xy_*), when the signal channel is responsible for the largest inter-regional correlations, then CC1 will be well-aligned to reveal changes in signal, *D*_CC1_ will approach optimal, and *R*_CC1_ will indicate the strength of the signal. Note that the interpretation of *R*_CC1_ depends on the strength of *C_xy_*.

In agreement with our theory and this working hypothesis, we found that the amount that *D*_CC1_ deviates from optimal decoding increased as *C_xy_* increased for both the visual system (Fig. 4B and C) and olfactory system (Fig. 4F and G). For this result, we excluded cases with low *R*_CC1_ (Materials and methods), because our theory predicts that very low *R*_CC1_ precludes high-quality CC1 decoding as discussed above. As a more direct test of the predicted dependence on *R*_CC1_ (Fig. 2D), we showed that CC1 decoding deviates further from optimal as *R*_CC1_ decreases (Fig. 4D, E, H, and I). Here, we excluded cases with high *C_xy_* (Materials and methods), because high *C_xy_* precludes high-quality CC1 decoding. We also performed a multivariate linear regression to predict deviation from optimal decoding using *C_xy_* and *R*_CC1_. The regression coefficients for *C_xy_* were positive and significant; the coefficients for *R*_CC1_ were negative and significant (Fig. S3). Thus, we conclude that, for diverse types of sensory stimulation in multiple mice, there are many neural populations that exhibit near-optimal CC1 decoding. Moreover, the populations that deviate from optimal CC1 decoding are consistent with our theory; they behave as if they are involved in other interactions between cortex and upstream sensory regions that are unrelated to the sensory signals we are decoding.

What about larger neural populations and canonical components beyond the first component? Indeed, our theory holds for arbitrarily large populations; our choice to work with 2 × 2 populations in Fig. 2 was primarily for simplicity and convenience of visualization. We repeated some of our analyses for 3 × 3 and 4 × 4 populations. In both cases, we found strong evidence that CC1 decoding stands up to 10-fold cross-validation and even reaches decoding accuracies slightly higher than those found in the 2 × 2 populations (Fig. S2). To find 3 × 3 and 4 × 4 populations with effective CC1 decoding, we selected neurons that performed well at the 2 × 2 level (those with *D*_CC1_ > 0.7). This mathematically ensures that the lower bound on optimal decoding accuracy for these larger populations was 0.7 because the optimal decoder could always project responses onto the same line that was optimal for the 2 × 2 case. However, in line with our theory and the hypothesis sketched in Fig. 4A, CC1 decoding did not always reach this optimal decoding level. As shown in Fig. 4J–L, deviations from optimal for 3 × 3 populations in the visual system were well explained by the same considerations of *C_xy_* and *R*_CC1_ that we described above for 2 × 2 populations. Finally, we note that we did not find any signs of effective CC2 decoding for the 3 × 3 case (Fig. 4J–L). Additional studies are needed to fully explore possibilities of decoding using higher canonical components, but our initial steps in this direction suggest that, at least for simple stimuli like static gratings, CC1 represents the best decoding subspace.

## Discussion

We have described an algorithm—CC1 decoding—that may be used by neural circuits in the primary sensory cortex to improve the accuracy of sensory decoding, in an “unsupervised” way, i.e. without “ideal observer” knowledge of stimulus labels. The algorithm achieves this decoding improvement by projecting high-dimensional population responses onto a 1D subspace with reduced noise (the first CC1). This subspace is readily found by CCA, provided that noise correlations between cortex and upstream brain regions are not too large and the signals are not too weak. We demonstrated that many real neurons measured in awake mice, in visual and olfactory systems (LGN and V1, OB and PC), meet the conditions required for CC1 decoding. We demonstrated these findings using populations of 4, 6, and 8 neurons from cortex and upstream brain regions. However, we emphasize that our theory predicts that CC1 decoding should work well with larger populations as well.

Before discussing further CC1 decoding, we note that there are alternatives to our interpretation of noise correlations as detrimental to coding. For example, one recent study showed that neurons in task-related neurons in V1 and middle temporal region exhibit more shared variability than task-independent neurons (40). The authors proposed that this shared variability could be useful for “tagging” which neurons are encoding task information and could aid in decoding sensory signals involved in the task. It remains unclear if this scheme might also be present for LGN and V1 or for OB and PC, but nonetheless, it stands as an interesting alternative to the age-old view of shared noise impeding encoding.

**Fig. 5.**
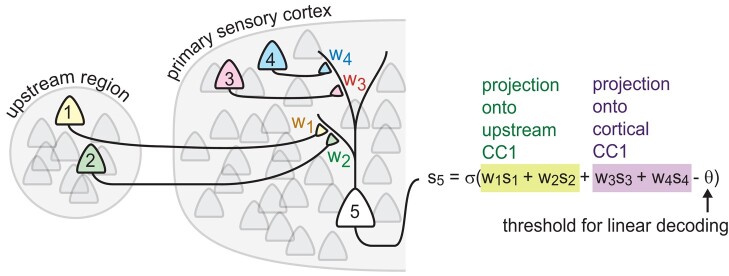
Biophysical implementation of CC1 decoding. Synaptically weighted summation of inputs naturally performs projections of high-dimensional input onto 1D subspace. The spike threshold ϴ naturally imposes a “decision boundary” for classifying stimulus type of input. Synaptic plasticity mechanisms can tune the synaptic weights so that the projections are aligned with CC1 (38).

How might real neurons implement the CC1 decoding algorithm we describe here? Assuming that there are some neurons with sufficiently small cross-region noise correlations (as we found in our experimental data), such an implementation requires three operations. First, the CC1 subspace must be identified. Second, neural activity must be projected onto CC1. Third, the projected activity must be thresholded to “decide” which type of stimulus was present. The latter two operations are quite naturally performed by neurons. Neurons sum up their inputs, weighted by synapse strength, which performs projections onto subspaces. For instance, in the cartoon in Fig. 5, neurons 3 and 4 together generate a 2D input signal to neuron 5, but when added up and weighted by w3 and w4, their input becomes 1D; it is projected onto a line determined by the relative weights of w3 and w4. Moreover, if neuron 5 also receives input from the upstream population (neurons 1 and 2 in Fig. 5), then its input could sum up two projections, one onto CC1 for the upstream population, and the other onto CC1 for the cortical population. Recalling that these two projections are maximally correlated, their sum would be an excellent 1D signal for decoding the stimuli. After this projection, the spiking mechanism of neurons naturally thresholds the 1D input. How might neurons identify CC1? In other words, how might they tune the synaptic weights so that they implement the specific projections onto CC1? A line of theoretical research has addressed this possibility directly (41–43) culminating recently (42) in a model with biologically plausible synaptic plasticity mechanisms. According to this theory, a network of reciprocally connected excitatory and inhibitory neurons that receive input like that received by neuron 5 in Fig. 5 can perform precisely the sum of two CC1 projections illustrated in Fig. 5. This operation can be performed “on-the-fly”; it does not require a “memory” of previous observations to be stored for later use. The synaptic plasticity rules are based solely on information available locally to each synapse—information about the pre- and postsynaptic neurons’ activity. Moreover, the plasticity rules are based on non-Hebbian mechanisms involving Ca^2+^ plateau potentials (44) which are consistent with evidence from the cortex. Taken together with our work here, we conclude that CC1 decoding not only can reach optimal limits of decoding but is also plausibly performed by cortical circuits.

Here, we focused on sensory coding subspaces, but our findings suggest a more general principle for multiplexing many functions within the same neural circuit. Any two brain regions that cooperate to execute a particular function are likely to exhibit some correlated activity. But this shared signal is likely mixed in with other activity (“noise”) that is involved in other ongoing functions. Our results suggest that the common, correlated activity between the two regions can define a CC1 subspace which effectively separates the function of interest from other ongoing functions, thus allowing the same circuits to execute many functions simultaneously.

## Materials and methods

### Experiments

The olfactory dataset was recorded and first reported by Bolding and Franks (45). Their methods were approved by Duke University Institutional Animal Care and Use Committee. Their methods for olfactory stimulation, head-fixation, respiration monitoring, electrophysiology, and spike-sorting were also described in detail previously (45). Here studied the following recordings: 170,608, 170,609, 170,613, 170,615, 170,618, 170,619, 170,621, 170,622. The visual dataset was first reported by Siegle et al. (16) recorded by the Allen Institute for Brain Science. The visual stimulation, head-fixation, electrophysiology, and spike-sorting are described and the data is available for public download Animal use protocols were approved by the Allen Institute's Institutional Animal Care and Use Committee. Here we analyzed experiments with the following session IDs: 754312389, 715093703, 750749662, 754829445, 757970808, 759883607, 761418226, 763673393, 799864342.

### CCA with Matlab

To perform CCA on experimental data, we used the Matlab function “canoncorr,” e.g. (*A*, *B*, ∼, *U*, *V*, ∼) = canoncorr(Region1Data, Region2Data). Here, each set of responses (e.g. Region1Data and Region2Data) is a *T* × *N* matrix (*T* trials, *N* neurons). The *i*th trial from Region1Data projected onto the *j*th CC is stored as *U*(*i*, *j*), and the vector that defines the direction of the *j*th CC in Region1 is stored as the *j*th column of A. Note that for the analytical study (Figs. 1 and 2), CCA was not done with Matlab; the CC directions were computed through the associated eigenvalue problem (see below and supplementary material).

### Optimal decoding

For 2D Poisson-distributed spike count responses, like the experimental data we studied here, typical supervised machine learning algorithms (e.g. LDA and support vector machines) often do not find the optimal decoding accuracy. Thus, we made a brute-force algorithm that projects the responses onto a line and evaluates decoding accuracy (as described in next subsection), trying many possible angles of projection between 0 and *π* rad (in steps of *π*/200 rad). Optimal decoding accuracy is that of the projection with the maximum decoding accuracy of all these projections.

### Decoding accuracy

The decoding accuracy of various linear projections is reported in the main text (optimal *D*, *D*_CC1_, *D*_n3_). After responses were projected down to one dimension, the decoding accuracy was determined by trying every possible threshold between the minimum and maximum projected response. The fraction of correct classifications for the best threshold was reported as decoding accuracy. For the analytical study of 2 × 2 populations a different approach (independent of a number of trials) was taken (see below and supplementary material).

### *C_xy_* and *R*_CC1_: definitions and details

*R*_CC1_ is defined as the Pearson correlation coefficient of responses $r_{CC1}^{1}$ and $r_{CC1}^{2}$, where $r_{CC1}^{i}$ is the 1D vector of responses from population *i* after projection onto its CC1 direction. By definition, CCA chooses the CC1 directions to maximize *R*_CC1_. *C_xy_* is the average pairwise noise correlation between the two populations. For each neuron, we calculate the mean response to all trials of type A and subtract it from each response to trials of type A. We repeat this for type B responses and for all neurons. After subtracting these stimulus-specific mean responses from each neuron, we then calculate the pairwise Pearson correlation coefficient for all neurons. *C_xy_* is defined as the average pairwise correlation of all cross-population pairs.

In Fig. 2C and D, we showed that *D*_CC1_ approaches optimal decoding for small *C_xy_* and large *R*_CC1_. To better separate the effects of these two quantities, we excluded cases with low *R*_CC1_ in Fig. 2C and excluded cases with high *C_xy_* for Fig. 2D. These cutoffs were *R*_CC1_ > 0.75 and *C_xy_* < 0.1. We did a similar cutoff for the results in Fig. 4, showing that *D*_CC1_ deviated from optimal for large *C_xy_* or small *R*_CC1_. For Fig. 4, the cutoffs were set as follows: for LGN-V1 data, *R*_CC1_ > 0.3 and *C_xy_* < 0.06; for OB-PC data, *R*_CC1_ > 0.4 and *C_xy_* < 0.1.

For the regression mentioned in the Results and summarized in Fig. S3, we employed a generalized least squares (GLS) method from the Python library *statsmodels* to fit the following regression equation: *y* = *β*_1_*x*_1_ + *β*_2_*x*_2_ + *ɛ*, where, *y* represents the dependent variable Δ, and *x*_1_ and *x*_2_ correspond to the independent variables *C_xy_* and *R*_CC1_, respectively. The GLS method estimates the coefficients, *β*1, and *β*2 that minimize the sum of squared residuals, accounting for potential heteroscedasticity in the error term *ɛ*.

### Selecting 2 × 2, 3 × 3, and 4 × 4 populations

The majority of our results are demonstrated with four neuron populations (two from cortex and two from upstream region). Ten thousand of these 2 × 2 populations were chosen at random to generate the results in Fig. 3C and H. In Fig. 4, the 10,000 populations were winnowed down to include only those with top 50 values of optimal decoding. In Figs. 3 and S2, we present results from ∼700 3 × 3 populations and 400 4 × 4 populations. These populations were selected from among neurons that performed well at the 2 × 2 level (the top 1,000 values of *D*_CC1_ from all 10,000 populations, considering only decoding of 0° vs. 90° gratings).

### Analytical 2 × 2 study

In Figs. 1 and 2, we consider the case of two populations (*X* and *Y*) of simulated neurons whose responses to two stimuli, *A* and *B*, are correlated both within and across populations. We assume the responses ( $r_{X}$ and $r_{Y}$) to each stimulus can be described by a multivariate Gaussian


$$P(r_{X},r_{Y}|S)=N\left(\left[\begin{array}{c} \mu_{X,S}\\ \mu_{Y,S} \end{array}\right],\Sigma_{S}\right),$$

where S={A,B}, $\mu_{X,S}\in R^{m}$, $\mu_{Y,S}\in R^{n}$, and $\Sigma_{S}$ is a symmetric, positive-definite matrix of size (m+n)×(m+n). Here populations *X* and *Y* contain *m* and *n* neurons, respectively (*m* = 2 and *n* = 2 for the simulated results in Figs. 1 and 2); for example, *Y* may be a cortical region and *X* may represent OB or LGN. Without loss of generality, we simplify notation by shifting the mean responses so that $\mu_{X,A}=0,\mu_{Y,A}=0$; thus, we can drop the stimulus subscript on the mean vectors and use $\mu_{X}=\mu_{X,B},\mu_{Y}=\mu_{Y,B}$. We further assume that the stimulus-conditioned noise covariance matrix is the same for each stimulus: i.e. that $\Sigma_{A}=\Sigma_{B}\;=:\Sigma$. For simplicity, we assume that noise correlations $c_{XY}$ were equal for any pair of cells across the two populations.


$$\Sigma =\Lambda \left[\begin{array}{cccc} 1 & c_{OB} & c_{XY} & c_{XY}\\ c_{OB} & 1 & c_{XY} & c_{XY}\\ c_{XY} & c_{XY} & 1 & c_{PC}\\ c_{XY} & c_{XY} & c_{PC} & 1 \end{array}\right]\Lambda ;\;\;\Lambda =\left[\begin{array}{cccc} \sigma_{X_{1}} & 0 & 0 & 0\\ 0 & \sigma_{X_{2}} & 0 & 0\\ 0 & 0 & \sigma_{Y_{1}} & 0\\ 0 & 0 & 0 & \sigma_{Y_{2}} \end{array}\right]$$

As noted earlier, mean response to stimulus *A* was 0, and mean response to stimulus *B* was:


$$\mu ={\left[\begin{array}{cccc} \mu_{X_{1}} & \mu_{X_{2}} & \mu_{Y_{1}} & \mu_{Y_{2}} \end{array}\right]}^{T}$$

Each of the 11 parameters $\mu_{X_{1}},\mu_{Y_{1}},\mu_{X_{2}},\mu_{Y_{2}},\sigma_{X_{1}},\sigma_{X_{2}},\sigma_{Y_{1}},\sigma_{Y_{2}},c_{OB},$$c_{PC},c_{XY}$ was chosen randomly from the following distributions:


$$\begin{array}{rl} & \sigma_{X_{1}},\sigma_{X_{2}},\sigma_{Y_{1}},\sigma_{Y_{2}}∼N_{F}(0,2)\\ & \mu_{X_{1}},\mu_{Y_{1}}∼N(0,1);\mu_{X_{2}},\mu_{Y_{2}}∼N_{F}(0,1)\\ & c_{OB},c_{PC},\tilde{c}∼U(0,1);c_{XY}=\max(\tilde{c}-0.01,0) \end{array}$$

Here, $N_{F}$ is the folded normal distribution (if X∼N(μ,σ),$\mathrm{then}\;|X|∼N_{F}(\mu ,\sigma )$), and *U* is the uniform distribution. Parameters are defined so that—without loss of generality— $\mu_{X_{2}},\mu_{Y_{2}}\geq 0$, all noise correlation parameters are non-negative, and $c_{XY}$ has about a 1% chance of being 0. For a single simulation, each parameter was chosen independently from the above distributions; the covariance matrix was then checked for positive definiteness (equivalently, $c_{XY}<\sqrt{\left(\left(1+c_{OB})(1+c_{PC}\right)\right)}/2$): if it failed, a new set of parameters was chosen. This was then repeated 50,000 times, allowing a robust and wide-ranging survey of possible signal and noise correlation structures.

We next explored decoding under different assumptions. First, we sought to determine how well the stimulus can be decoded from responses within each population. In this simplified setting (responses are Gaussian, and the noise covariance is stimulus independent), the optimal decoder is linear and can be determined by a simple analytical formula (see Supplementary material, Materials and methods). That is, we decode the stimulus by projecting the population response onto a single vector and then compare that value with a threshold. Next, we use the principal direction from CCA, or CC1, as a linear decoder. Finally, we artificially remove cross-population noise correlations by setting $c_{XY}=0$, and recompute the CCA with the revised stimulus-unconditioned covariance. We show that in this setting, the most correlated direction CC1 is in fact equal to the optimal projection vector (see Supplementary material, Materials and methods). These three decoding vectors—optimal, CC1, and CC1 with $c_{XY}=0$—are demonstrated in Fig. 5A as black, yellow, and green, respectively.

We computed the single-population optimal decoding directions $v_{X},v_{Y}$ using Eq. S1 and found the decoding accuracy by integrating the resulting 1D Gaussians (see Eq. S2). Similarly, we calculated the CC1 for each population using Eq. S4, and found the decoding accuracy using $v_{X,CC1},v_{Y,CC1}$ as projection vectors. To compute single-cell decoding accuracy, we integrated under the marginal distributions given by projecting onto the coordinate directions $e_{1}=\left[\begin{array}{c} 1\\ 0 \end{array}\right],\;e_{2}=\left[\begin{array}{c} 0\\ 1 \end{array}\right]$.

## Supplementary Material

pgae010_Supplementary_Data

## Data Availability

Data analysis code is available without restriction on Figshare: https://doi.org/10.6084/m9.figshare.24802944.v1. All experimental data analyzed here is currently posted on freely accessible repositories. The visual system electrophysiology dataset is freely available from the Allen Institute at https://allensdk.readthedocs.io/en/latest/visual_coding_neuropixels.html, and the olfactory system electrophysiological dataset is freely available from Collaborative Research in Computational Neuroscience data sharing website: https://doi.org/10.6080/K00C4SZB.

## Preprint

This manuscript was posted as a preprint on bioRxiv: https://doi.org/10.1101/2022.06.15.496327.
