# Supplementary material for: Sensory input to cortex encoded on low-dimensional periphery-correlated subspaces
Source: PNAS Nexus. 2024 Jan 10;3(1):pgae010. doi: 10.1093/pnasnexus/pgae010 (PMC10798852; doi:10.1093/pnasnexus/pgae010)
Supplement: pgae010_Supplementary_Data [file pgae010_supplementary_data.pdf]

Supporting Information for

Sensory input to cortex encoded on low-dimensional periphery-correlated subspaces

Andrea K Barreiro, Antonio J Fontenele, Cheng Ly, Prashant C Raju, Shree Hari Gautam, Woodrow L Shew

Corresponding Author: Woodrow L Shew
E-mail: shew@uark.edu

This PDF file includes:

Supporting text
Figs. S1 to S3
SI References

Supporting Information Text

Supplemental Experimental Results

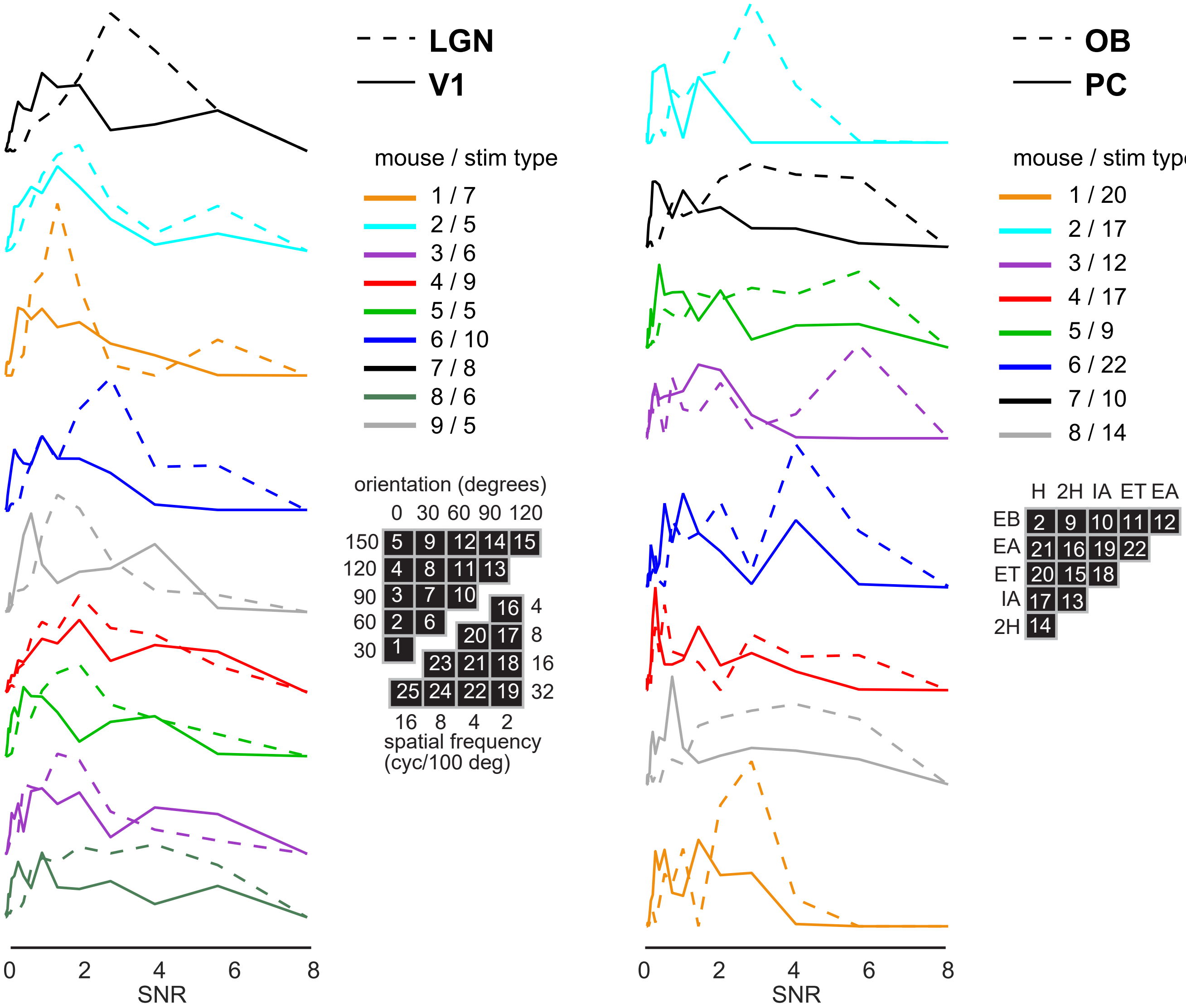

Fig. S1. Signal-to-noise ratio is lower in cortex than upstream regions. In the main text, it was suggested that signal-to-noise is lower in primary sensory cortex compared with the upstream regions, closer to the sensory periphery, that provide sensory input to cortex. Here we show quantitatively that this is true for the visual system and olfactory system data that we analyzed. [Left] Each solid line is a distribution of SNR values for the 10000 randomly chosen pairs of V1 neurons analyzed in the main text. Each dashed line is a SNR distribution for the corresponding 10000 pairs of LGN neurons. Here we took the 2x2-population-level SNR to be $\sqrt{\mu^T \Sigma^{-1} \mu}$, where μ is a two-element vector of response differences (response to stimulus type A minus response to stimulus type B) and Σ is the response covariance matrix for the two neurons. Each color represents an example from a different mouse and a different pair of stimuli. The 'stim type' number in the legend refers to a specific pair of grating orientations or spatial frequencies as labeled in the black grids. [Right] Same as the left, but based on the olfactory system. Note that for both the visual and the olfactory systems, the solid distributions indicate a lower typical SNR in cortex compared to the upstream regions (dashed). Also note that this set of example mice and stimulus types is the same as those shown in Fig 4B,D,F and H, with the same color code.

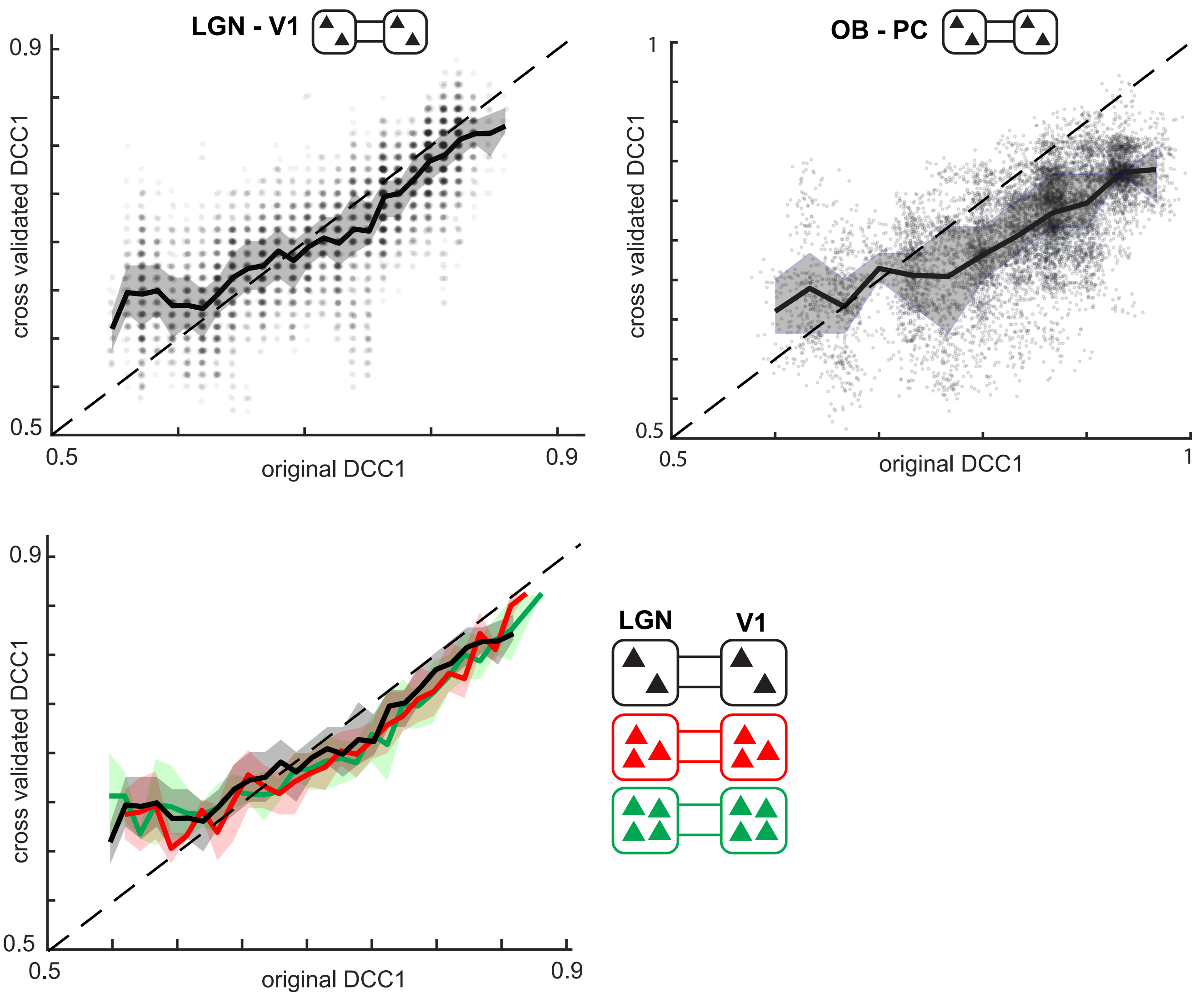

Fig. S2. Cross-validation of DCC1. Like many other decoding algorithms, our proposed CC1 decoding can generate artificially high values of decoding accuracy if there are too few stimulus trials or if response dimensionality is too high. In the context of our work, response dimensionality is simply the number of neurons from each population. To account for this possibility, we did a 10-fold cross validation to verify that our measured values of DCC1 were reliable. For a given 2x2 population, we used 9/10 of the stimulus trials to calculate CC1 directions and determine the optimal decoding threshold on the CC1-projected responses. Then, we used the same projection and threshold to calculate decoding accuracy for the held out 1/10 of trials. We repeated this for the 10 unique folds of training and hold-out trials and finally averaged the 10 decoding accuracy values across the 10 folds. These averaged decoding accuracies are reported here compared to the original decoding accuracies reported in the main text. Note that the cross validated DCC1 values are strongly correlated with the original values, which means they are reliable. However, it is clear that for the olfactory data (right), which had only 15 trials per stimulus type, original DCC1 is more prone to over estimation compared the cross validated DCC1. The visual system data (left) included 42 trials per stimulus type, which resulted in less bias, i.e. a better match between original and cross validated DCC1. The dashed line (slope unity) marks equality between original and cross validated DCC1. Each point represents one of 10000 randomly chosen populations. The dark line and shaded area indicates the median and quartiles of the points, respectively. Points are displayed with 20% opacity, so that density of points is clearer. [Bottom panel] Finally, we performed a similar 10-fold cross-validation for 3x3 and 4x4 populations from the visual system (see Methods in main manuscript) and found reliable results like the 2x2 case.

2x2 populations

β1	β2	R-square
4.9+-0.7	-0.7+-0.2	0.6+-0.03

3x3 populations

β1	β2	R-square
6.1+-0.5	-0.7+-0.1	0.71+-0.02

Fig. S3. Regression results. For the regression mentioned in the Results, we employed a generalized least squares (GLS) method to fit the following regression equation: $y = \beta_1 x_1 + \beta_2 x_2 + \epsilon$, where, y represents the normalized deviation from optimal decoding (Δ in the main text), and x_1 and x_2 correspond to the independent variables Cxy and RCC1, respectively. Here we report the mean $\pm$ standard deviation across animals.

Mathematical Results

Introduction. We consider the case of two populations of neurons whose responses to two stimuli, A and B, are correlated both within and across populations. We assume the responses to each stimulus can be described by a multivariate Gaussian, i.e.

$$P\left(r_X,\ r_Y|S\right) = \mathrm{N}\left(\begin{bmatrix}\mu_{X,S}\\ \mu_{Y,S}\end{bmatrix}, \Sigma_S\right),$$

where $S = \{A, B\}, \mu_{X,S}\ \in \mathbb{R}^m, \mu_{Y,S}\ \in \mathbb{R}^n$, and Σ_S is a symmetric, positive-definite matrix of size $(m+n)\times(m+n)$. Here X and Y refer to two populations of neurons which are both responsive to A and B, containing m and n neurons respectively; for example, Y may be a cortical region and X a pre-cortical region which supplies afferent input to Y. Without loss of generality, we simplify notation by shifting the mean responses so that $\mu_{X,A} = 0$, $\mu_{Y,A} = 0$; thus, we can drop the stimulus subscript on the mean vectors and use $\mu_X = \mu_{X,B}$ and $\mu_Y = \mu_{Y,B}$.

We next consider how to decode the stimulus, using only the responses within each population. We assume that the covariance matrix is the same for both stimuli: i.e. $\Sigma_A = \Sigma_B =: \Sigma$. In this case the optimal decision boundary is given by a hyperplane in $\mathbb{R}^m$ or $\mathbb{R}^n$; equivalently, by a one-dimensional projection of the response vector. The decision boundary is given by (for example) $u \in \mathbb{R}^m$ such that $u^T\Sigma_X^{-1}\mu_X = \frac{1}{2}\mu_X^T\Sigma_X^{-1}\mu_X + \log\frac{P(B)}{P(A)}$, (Here, Σ_X and Σ_Y are the marginal covariances in populations X and Y respectively.) Therefore, the projection vector must be the normal vector to this plane; i.e.:

$$v_X = \Sigma_X^{-1}\mu_X, \quad v_Y = \Sigma_Y^{-1}\mu_Y \qquad [1]$$

in populations X and Y respectively. Alternatively, observing that $v^T r_X|S$ is a one-dimensional Gaussian with

$$\mathbb{E}[v^T r_X|S] = v^T\mu_{X,S}, \quad \mathrm{Var}[v^T r_X|S] = v^T\Sigma v, \qquad [2]$$

we can derive the same outcome by maximizing the signal-to-noise ratio; i.e. $v_X = \mathrm{argmin}\left(\frac{\sqrt{v^T\Sigma v}}{v^T\mu_X}\right)$. From the perspective of *linear discriminant analysis*, this maximizes between-class (where "class"=stimulus identity) variability while minimizing within-class variability (1).

When cross-region noise correlations are absent, CC1 is a perfect decoder. We now compute the projection directions associated with canonical correlation analysis (CCA). Given two sets of zero-mean observations from X and Y, the goal of CCA is to find the linear projections of the observations that are maximally correlated (2). This technique uses the full stimulus-averaged population response; however, we will show that under certain conditions, the maximally correlated direction from CCA coincides with the optimal decoder. Assuming $P(A) = P(B)$, the covariance structure within each population is

$$\Sigma_{XX} = \frac{1}{4}\mu_X\mu_X^T + \Sigma_X;\ \Sigma_{YY} = \frac{1}{4}\mu_Y\mu_Y^T + \Sigma_Y$$

while the stimulus-averaged covariance matrix between populations X and Y is

$$\Sigma_{XY} = \frac{1}{4}\mu_X\mu_Y^T + \Sigma_C \qquad [3]$$

Here Σ_X, Σ_Y, and Σ_C are the covariances within and across-populations: i.e.

$$\Sigma = \begin{bmatrix}\Sigma_X & \Sigma_C\\ \Sigma_C^T & \Sigma_Y\end{bmatrix}$$

We now seek to find the directions which maximize correlation across the population; that is

$$R_{CC1} = \max_{\mathbf{a},\mathbf{b}} \frac{\mathbf{a}^T\Sigma_{XY}\mathbf{b}}{\sqrt{\mathbf{a}^T\Sigma_{XX}\mathbf{a}}\sqrt{\mathbf{b}^T\Sigma_{YY}\mathbf{b}}}$$

We denote the vectors that achieve this maximum as $v_{X,CC1}$ and $v_{Y,CC1}$ respectively; i.e.

$$v_{X,CC1}, v_{Y,CC1} = \mathrm{argmax}_{\mathbf{a},\mathbf{b}} \frac{\mathbf{a}^T\Sigma_{XY}\mathbf{b}}{\sqrt{\mathbf{a}^T\Sigma_{XX}\mathbf{a}}\sqrt{\mathbf{b}^T\Sigma_{YY}\mathbf{b}}}$$

The vectors $v_{X,CC1}$ and $v_{Y,CC1}$ can be obtained by finding the principal eigenvectors of D_X and D_Y respectively:

$$D_X = \Sigma_{XX}^{-1}\Sigma_{XY}\Sigma_{YY}^{-1}\Sigma_{XY}^T;\quad D_Y = \Sigma_{YY}^{-1}\Sigma_{XY}^T\Sigma_{XX}^{-1}\Sigma_{XY} \qquad [4]$$

and the corresponding eigenvalue is the correlation (R_{CC1}) squared: that is,

$$D_X v_{X,CC1} = \lambda v_{X,CC1} \Leftrightarrow R_{CC1} = \sqrt{\lambda}$$

We note that the cross-covariance matrix Σ_{XY} has two contributions, one reflecting signal correlations ($\frac{1}{4}\mu_X\mu_Y^T$) and the other noise correlations (Σ_C). The latter reflects trial-to-trial correlations which are not reflected in the mean response. We will now show that when noise correlations are absent ($\Sigma_C = 0$), the principal CCA direction coincides with the optimal decoding direction. Without loss of generality, we focus on D_X; parallel statements hold for D_Y.

Lemma 1: If $\Sigma_C = 0$, then D_X is a rank 1 matrix.

Proof. It is well known that the rank of a matrix product is bounded above by the minimum rank of the matrices; i.e. $\text{rank}(AB) \leq \min(\text{rank}(A), \text{rank}(B))$. From Eq. (3) Σ_{XY} is the sum of two matrices, the first of which is rank 1; if $\Sigma_C = 0$, therefore, the sum is rank 1 as well. Therefore any matrix product that includes Σ_{XY} has rank at most 1. □

Theorem 1: If $\Sigma_C = 0$, then the correlated (non-zero) eigenvector of D_X coincides with the projection direction which is optimal for decoding.

Proof. Recall that Eq. (1) shows that $v_X \propto \Sigma_X^{-1}\mu_X$. We will show that v_X is also an eigenvector of D_X.

Consider the formula for D_X:

$$D_X = \Sigma_{XX}^{-1}\Sigma_{XY}\Sigma_{YY}^{-1}\Sigma_{XY}^T$$

The cross-population correlation matrix Σ_{XY} is rank 1 and $\text{range}(\Sigma_{XY}) = \text{Span}\,\{\mu_X\}$. Therefore, $\text{range}(D_X) = \text{Span}\left\{\Sigma_{XX}^{-1}\mu_X\right\}$.

Next, we write Σ_{XX}^{-1} in terms of Σ_X^{-1}. Using the matrix determinant lemma, and noting that

$$\Sigma_{XX} = \Sigma_X + \mathbf{u}\mathbf{u}^T$$

where $\mathbf{u} = \mu_X/2$,

$$\Sigma_{XX}^{-1} = \Sigma_X^{-1} - \frac{\Sigma_X^{-1}\mathbf{u}\mathbf{u}^T\Sigma_X^{-1}}{1 + \mathbf{u}^T\Sigma_X^{-1}\mathbf{u}} \qquad [5]$$

$$= \Sigma_X^{-1} - \Sigma_X^{-1}\mathbf{u}\left(\frac{\mathbf{u}^T\Sigma_X^{-1}}{1 + \mathbf{u}^T\Sigma_X^{-1}\mathbf{u}}\right) \qquad [6]$$

The second term *already* maps into $\text{Span}\left\{\Sigma_X^{-1}\mu_X\right\}$, regardless of what vector is multiplied on the right. In conclusion, $\text{range}(D_X) = \text{Span}\left\{\Sigma_X^{-1}\mu_X\right\}$; i.e. $D_X v_X \propto v_X$. □

By using Eq. (6) (and the analogous simplification for Σ_{YY}^{-1}), one can confirm that the corresponding eigenvalue is

$$\lambda = \left(\frac{s_X^2}{4 + s_X^2}\right)\left(\frac{s_Y^2}{4 + s_Y^2}\right) \qquad [7]$$

where $s_X = \sqrt{\mu_X^T\Sigma_X^{-1}\mu_X}$ and $s_Y = \sqrt{\mu_Y^T\Sigma_Y^{-1}\mu_Y}$ are the signal-to-noise ratios for the X and Y populations respectively.

Theorem 2: If $\Sigma_C = 0$, then any other eigenvector of D_X gives chance-level decoding.

Proof. If $\mathbf{v}^T\mu_X = 0$, then $\Sigma_{XY}^T\mathbf{v} = 0$ and therefore $D_X\mathbf{v} = 0$. Therefore $\mathbf{v}$ is an eigenvector of D_X with eigenvalue 0. But then

$$E[\mathbf{v}^T r_X | A] = E[\mathbf{v}^T r_X | B] = 0$$

i.e. the stimuli A and B cannot be discriminated. □

References

1. J Cunningham, Z Ghahramani, Linear dimensionality reduction: Survey, insights, and generalizations. *J. Mach. Learn. Res.* **16**, 2859–2900 (2015).
2. H Hotelling, Relations between two sets of variates. *Biometrika* **28**, 321–377 (1936).
